# Impact of a Dietary Citrus Extract on the Behavior and Production of Lactating Dairy Cows Following Regrouping: A Preliminary Study

**DOI:** 10.3389/fvets.2021.773399

**Published:** 2022-01-20

**Authors:** Felipe H. Padua, Kaitlyn M. Dancy, Renée Bergeron, Trevor J. DeVries

**Affiliations:** Department of Animal Biosciences, University of Guelph, Guelph, ON, Canada

**Keywords:** citrus extract, social stress, welfare, grooming, agonistic interaction, behavior

## Abstract

Exposure of lab animals, humans, and pigs to olfactory sensory feed additives may reduce response to stress and anxiety. The objective of this preliminary study was to determine if feeding a citrus-based olfactory sensory functional feed extract (derived from *Citrus sinensis*) reduces the negative impact of regrouping of lactating dairy cows. Thirty-two (parity = 2.0 ± 1.2; mean ± SD), mid-lactation Holstein dairy cows (169.8 ± 16.8 DIM) were enrolled as focal cows in this study and housed individually in a tie-stall facility where they were randomly assigned to 1 of 2 treatment diets: (1) control total mixed ration (TMR) (control; *n* = 16; primiparous = 7; multiparous = 9), or (2) control TMR with 4 g/d of citrus extract (CE) (Phodé, Terssac, France) (CE; *n* = 16; primiparous = 7; multiparous = 9). Cows were fed their experimental diets for 7 d in the tie-stall facility (baseline), then moved to 1 of 2 experimental free-stall pens (containing 29 other cows) for a period of 7 d, where they remained on the same treatment diet as before. Compared with their baseline, primiparous control cows had decreased rumination time on d 1 and 2, had decreased lying time on d 1, and tended to have decreased lying time on d 2 and 3 following regrouping. In contrast, primiparous cows fed the CE diet did not experience a change in rumination and lying time. Primiparous CE cows had greater feeding time on d 1 and tended to have greater feeding time on d 2 after regrouping compared to primiparous control cows. Primiparous control cows had greater idle standing time, as compared to the CE cows, across the 7 d after regrouping. Primiparous CE cows initiated less total competitive behavior after regrouping, but were also displaced more frequently from the feed bunk and from the free stalls on d 1 after regrouping, as compared to the primiparous control cows. For multiparous cows, CE supplementation was not consistently associated with any benefits to behavior or production after regrouping, possibly because these cows were more experienced with social stressors. Results indicate that feeding CE to mid-lactation naïve primiparous dairy cows may reduce the initiation of competitive interactions and lessen the reduction in rumination and lying time after regrouping. These results need to be verified in further studies where potential confounding effects (e.g., pen social dynamics, pen location) are minimized.

## Introduction

Dairy cows in commercial farms are frequently moved to form groups similar in age, stage of lactation, milk production, health, and reproductive status (1, 2). It has been reported that cows may experience up to 4–5 regrouping events during any single lactation (1).

Researchers have previously suggested that mixing cows with unfamiliar animals, which already have an established social order, destabilizes the social dynamic within the group (2). After regrouping, dairy cows increase their level of physical (e.g., threats, butts, grooming) and non-physical interactions to re-establish social relationships (2, 3). Any increases in competitive behavior may result in reduced lying, feeding, and rumination behavior, leading to transitory decreased dry matter intake (DMI) and milk production [e.g., (4–6)]. Torres-Cardona et al. (7) similarly demonstrated that relocation may reduce milk production on the day after relocation, with a greater impact on first-lactation heifers compared with mature cows.

Given the negative impacts of regrouping, and its elevated frequency within modern dairy farm management, ways to reduce these effects need to be identified. This may be done through changes in farm management. For example, Talebi et al. (8) demonstrated that the increases in competitive behavior in cows following regrouping and associated decreases in lying times may be mitigated by reducing pen stocking density. Further, Tesfa (9) demonstrated that lactating cows introduced into new groups of cows as pairs, as opposed to individually, experienced no drop in milk production as demonstrated in previous studies. More recently, Mazer et al. (10) demonstrated that primiparous cows, when individually moved to a new pen after calving, exhibited greater fecal cortisol metabolite concentration in the days subsequent to regrouping as compared with primiparous cows moved along with a partner. As noted by Mazer et al. (10), fecal cortisol metabolites may be used as a physiological measure of stress. Further, those researchers reported that greater fecal cortisol metabolite concentration in primiparous cows than multiparous cows when introduced to a new group individually, suggesting that primiparous cows may be more sensitive to the negative effects of regrouping as compared with multiparous cows (10, 11). Multiparous cows may be more experienced and familiar with the social stress associated with regrouping events and, thus, experience fewer negative effects after regrouping as compared to primiparous cows who would be more naïve to such events.

There may be other opportunities to reduce the negative impacts of regrouping, including the use of olfactory sensory feed additives. Exposure to citrus extract (CE) essential oil, derived from *Citrus sinensis* (“sweet orange”), has been demonstrated to reduce anxiety in male Wistar rats (12) and reduce mean blood pressure, respiratory rate, and pulse rate in children during a stressful situation (13). Similarly, Menneson et al. (14) demonstrated that pigs that exposed to CE had a lesser stress response when injected with a substance to induce stress than pigs not exposed to CE. Perhaps most compellingly, Lehrner et al. (15) observed that, in a study of men and women, diffusing *C. sinensis* essential oil into a dental office waiting room reduced self-reporting of anxiety and increased reporting of factors associated with positive mood (*n* = 50 subjects) compared with a control (no scent; *n* = 51 subjects). The mode of action of CE is hypothesized to be through olfactory stimulation of the brain. Coutens et al. (16) demonstrated that a *C. sinensis*-based olfactory ingredient exerted anti-stress effects in a mouse model, not only in healthy animals, but also in those subjected to chronic stress. Those researchers further demonstrated that those beneficial effects were attenuated by applying a pharmacological agent capable of transiently blocking olfaction. Moreover, it was demonstrated that CE provision was accompanied by an increase in the maturation process of neurons in the hippocampal dentate gyrus (16), thus reinforcing the links between the olfactory system and this brain region strongly involved in emotions. In support of that, Val-Laillet et al. (17) demonstrated that feeding pigs a *C. sinensis* based CE feed additive had positive impacts on brain activation in the insular cortex, the amygdala, and the striatum (putamen and caudate), suggesting that the CE stimulated reward perception and anticipation in pigs. Finally, an imaging study in pigs treated with a *C. sinensis* based CE feed demonstrated that olfactory stimulation induced by this ingredient increases the activity of several brain regions associated with the regulation of cognitive and emotional processes (14).

To our knowledge, no research to date has been conducted to evaluate the effects of a *C. sinensis* based olfactory sensory functional food ingredient on the response of dairy cattle to a potentially stressful situation. Thus, the objective of this study was to determine if feeding a citrus-based olfactory sensory functional feed extract (derived from *C. sinensis*) reduces the negative impact of social regrouping of lactating dairy cows on behavior and milk production. It was hypothesized that cows supplemented with CE in their diet would experience fewer negative effects on their behavior (feeding, ruminating, lying, and social) and milk production after being moved into a new group of cows, compared with cows fed a control diet. Additionally, it was hypothesized that CE supplementation would have a greater beneficial effect on more naïve, primiparous cows as compared with multiparous cows, who would have had more previous experience with social stressors.

## Materials and Methods

### Animals and Housing

Thirty-two lactating Holstein dairy cows, including 14 primiparous and 18 multiparous (CE; parity = 2.8 ± 1.0 and Control; parity = 2.8 ± 1.0, mean ± SD), were randomly selected from the University of Guelph, Elora Research Station—Ontario Dairy Research Center (Elora, Ontario, Canada) dairy herd for use in this study. At the time of entry into the study, selected multiparous and primiparous cows were in mid-lactation, 163.0 ± 10.0 and 178.6 ± 20.3 (mean ± SD) DIM, respectively, and were producing on average 40.3 ± 5.5 and 32.2 ± 4.2 kg/d of milk, respectively. Cows used in this study were kept in one of two identical free-stall pens (except for during the acclimatization period, see below), each containing 30 free-stalls (cubicles) and two water troughs. Stalls were mattress-based (Pasture Mat; ProMat, Woodstock, ON, Canada) and bedded with chopped straw. The two free-stall pens were 56.4 m apart. Each free-stall pen contained a stationary (non-mechanical) scratching brush. Cows were fed a total mixed ration (TMR) (Table 1), 1x/d, between 09:30 and 11:30 h. A feed refusal rate of 5% of offered feed was targeted. Cows were milked 2x/d (at 04:30 and 16:30 h). Animals were managed according to the standard operating procedures for this facility. All cows were routinely checked for health status, both prior to and throughout this study.

**Table 1:** Ingredient and chemical composition (mean ± SD) of the lactating cow diet^a^.

**Composition**	**CE**	**Control**
**Ingredient, % of DM**		
Corn silage^b^	29.3	
Wheat straw^c^	1.8	
Alfalfa haylage^d^	29.5	
High moisture corn^e^	25.3	
Lactating cow supplement^f^	14.1	
**Chemical composition^g^**		
DM, %	48.1 ± 1.40	48.2 ± 1.76
CP, % of DM	14.7 ± 0.67	14.7 ± 1.01
ADF, % of DM	20.4 ± 0.73	20.0 ± 0.98
DF, % of DM	29.3 ± 0.86	29.3 ± 1.40
TDN, % of DM	73.0 ± 0.57	73.3 ± 0.76
Starch, % of DM	28.4 ± 1.92	29.7 ± 2.04
Sugar, % of DM	3.5 ± 0.58	3.3 ± 0.24
NFC, % of DM	44.5 ± 0.60	44.5 ± 1.61
Ca, % of DM	0.9 ± 0.05	0.9 ± 0.05
P, % of DM	0.4 ± 0.04	0.4 ± 0.05
K, % of DM	1.4 ± 0.11	1.4 ± 0.14
Na, % of DM	0.4 ± 0.03	0.4 ± 0.04
Mg, % of DM	0.4 ± 0.02	0.4 ± 0.03
NE_L_, Mcal/kg of DM	1.7 ± 0.01	1.7 ± 0.02

a *CE, lactating cow diet with control TMR with 4 g/d of citrus extract; Control, lactating cow diet*.

b *Corn silage had a DM of 33.4%*.

c *Straw had a DM of 89.2%*.

d *Alfalfa haylage had a DM of 32.8%*.

e *High moisture corn had a DM of 74.0 ± 2.6% and chemical composition (DM basis) 8.0 ± 1.3% CP, 2.8 ± 0.4% ADF, and 8.5 ± 0.8% NDF*.

f *Supplied by Floradale Feed Mill Ltd. (Floradale, Ontario, Canada) including ingredients (as is); 40% Bypass Soybean meal, 29% soybean meal, 7% Canola meal, 5.6% Wheat shorts, 3% limestone calcium carbonate, 3.1% sodium sesquicarbonate, 3% fine salt, 2.8% monocalcium phosphate, 1.7% magnesium oxide, 1.4% Diamond V Yeast XP, 1% Tallow, 0.75% Integral, 0.7% Inorganic lactating mix, 0.5% Metasmart, 0.3% DCAD+, 0.04 Sulfur, and 0.05% Rumensin,. Lactating cow supplement had a DM of 89.3%*.

g *Values were obtained from chemical analysis of TMR samples. NE_L_ was calculated based on NRC (18) equations*.

The use of cows and experimental procedures complied with the guidelines of the Canadian Council on Animal Care (19) and were approved by the University of Guelph Animal Care Committee (AUP#4131).

### Experimental Design

Sample size and power analyses were used to calculate [as per (20)] the minimum number of replicates needed per treatment (*n* = 16) to detect a 10% level of observed mean difference for the primary outcome variables, including milk yield, lying time, rumination time, and feeding time. Estimates of variation for these variables (average CV = 14%) were based on values from previous regrouping studies (5, 6). We also calculated that for the number of primiparous (*n* = 7) and multiparous (*n* = 9) cows per treatment, mean differences of 15 and 13%, respectively, could be detected.

Focal cows (*n* = 32) were individually and randomly assigned, balancing for parity (i.e., number of primiparous and multiparous in both treatments) by the research coordinator of the facility (who was not blind to the treatments), to 1 of 2 treatments: (1) TMR (control diet; *n* = 16; primiparous = 7; multiparous = 9), or (2) TMR with 4 g/d of CE (Phodé, Terssac, France) (CE diet; *n* = 16; primiparous = 7; multiparous = 9). The CE additive required a carrier (wheat middlings) to ensure even distribution in the TMR. Thus, CE cows were fed the TMR with 200 g/cow/d of the feed additive (which included 196 g/cow/d of wheat middlings mixed with 4 g/cow/d of CE). To ensure consistency between diets, the control cows were fed a TMR with a placebo (200 g/cow/d of wheat middlings). To prevent cross-contamination of the treatments, the experimental diets were prepared and delivered from two different feed wagons (i.e., same make and model), while the distance between cows on different treatments was maximized. On d −7, focal cows were moved, 2 at a time (i.e., each week), from a non-experimental free-stall pen (different free-stall pen area than the experimental pens cows were later moved into) to a tie-stall area, where they were housed (and fed and milked) in tie-stalls for 7 d. This move was done to allow cows to acclimate to the treatment diets. In the tie-stall area, newly enrolled focal cows (one control and one CE) were at minimum 15 m (and maximum 21 m) apart from each other. Other non-experimental cows were occasionally housed in tie-stalls between the focal cows. Tie-stalls were mattress-based (Pasture Mat; ProMat, Woodstock, ON, Canada) and bedded with chopped straw. Upon entry to that housing (i.e., two cows each week), primiparous and multiparous cows were randomly assigned to 1 of the 2 experimental treatment diets and fed those diets for an adaption period of 7 d (21) in that tie-stall area. At this stage of the study, focal cows were not paired to move to the tie stall by parity, but rather by cow availability. Dry matter intake was recorded daily for these cows, based on feed offered and feed refused. After 7 d of feeding the treatment diets, focal cows were moved to one of the two experimental free-stall pens right before feeding time (at 10:30 h). At that time point each week, two more focal cows were moved into the tie-stall area. Cows in the experimental free-stall pens were fed the same diets as the cows that were moved into those respective pens [i.e., one pen was fed the control TMR with placebo (200 g/cow/d of wheat middlings), while the other pen was fed the control TMR with the feed additive mixture (196 g/cow per day of wheat middlings mixed with 4 g/cow/d of CE)]. Pen DMI were recorded 3 d per week based on feed offered and feed refused.

Prior to the first four focal cows being moved into the free-stall pens, cows in those pens were fed their respective treatment diets for a 2-week period to allow those cows establish social order and acclimate to those diets. Those pens each consisted of 30 lactating dairy cows in mid to late lactation. The composition of cows in those pens remained similar and consistent across the study. In the pen fed the CE diet, multiparous cows averaged (mean ± SD) 258.4 ± 76.1 DIM, 2.3 ± 0.8 lactations, 772.3 ± 87.8 kg of BW, 3.19 ± 0.3 body condition score (BCS), and in the pen fed the control diet multiparous cows averaged 252.0 ± 69.3 DIM; 2.7 ± 0.9 number of lactations, 767.6 ± 74.3 kg of BW, and 3.17 ± 0.3 BCS. Meanwhile, in the pen fed the CE diet, the primiparous cows averaged 278.4 ± 73.2 DIM, 744.8 ± 80.2 kg of BW, 3.3 ± 0.3 BCS and in the pen fed the control diet primiparous cows averaged 283.8 ± 76.0 DIM, 734.7 ± 68.7 kg of BW, and 3.2 ± 0.4 BCS. Upon entry of the experimental focal cows to those pens, one late lactation cow from each pen was randomly removed to make space for the incoming cow. The focal cows were monitored for a period of 7 d following regrouping in their new pen. After 7 d, the next two focal cows were moved into the free-stall pens, with again one late lactation cow from each group being removed to make space for the incoming cow. This process was repeated until all 16 cows per treatment had been introduced to the experimental free-stall pens. Overall, this study had a duration of 18 weeks, occurring from October 2019 to January 2020.

### Behavioral Data Collection

Standing and lying behavior data of the focal cows were recorded with electronic data loggers (HOBO Pendant G Data Logger, Onset Computer Corporation, Bourne, MA), as validated by Ledgerwood et al. (22). Measurements were taken at 1-min intervals; leg orientation data was used to compute standing and lying duration. Data loggers were attached to the medial side of the hind leg of each cow using veterinary bandaging tape (Vetrap Bandaging Tape, 3M, London, ON, Canada) at entry to the tie-stall barn area. After 7 d (at time of regrouping), loggers were removed (and data extracted), and a new logger was placed on the other hind leg for an additional 7 d, after which the data was extracted. Data from these loggers were used for the analysis comparing the focal cow lying time (min/d) after regrouping with their own baseline measured before regrouping.

An electronic monitoring system (HR-TAG-LD, SCR Engineers Ltd., Netanya, Israel), as validated by Schirmann et al. (23), was used in this study to monitor rumination activity of all focal cows. Rumination data loggers were attached to a nylon collar that was fitted to each cow on the first day of enrolment to monitor rumination activity throughout the study period. Data were continuously uploaded to a control unit through a radio frequency reader. Raw data were stored in 2-h intervals and then combined into a continuous record to determine the total time spent ruminating each day for each cow.

Feeding time, social behavior, and grooming behavior of focal cows were determined from continuous video recordings, captured by video cameras (YI Outdoor Security Camera 1080p; YI Technology, Shanghai, China). To identify the focal cows in the videos, pink veterinary bandaging tape (Vetrap Bandaging Tape, 3M, London, ON, Canada) was attached to the hind legs of the focal cows before they entered the free-stall pens. Cow markings were also photographed and used for identification in cases where the hinds legs were not visible on video. The videos were recorded on a 32GB microSD card (SanDisk Ultra microSD UHS-I; Milpitas, California, United States of America) and replaced every 2 d with another microSD card. The cameras were set to record at 20 frames/s and were positioned approximately 5 m above the pens such that each pen was fully visible from one camera.

Feeding behavior of the focal cows was recorded using instantaneous 10-min video scan sampling (as validated by 23) beginning the moment of being regrouped in the free-stall pen until d 7 after regrouping. For each scan, at 10-min intervals, a cow was considered feeding when her head was completely past the headlocks and over the feed. To calculate total time spent feeding (min/d), the number of scans per day where the cow was feeding was multiplied by 10 (24). To calculate daily idle standing time (min/d), time spent feeding (min/d), and lying (min/d) were subtracted from the total minutes of the day (1,440 min/d) (25). Idle standing time included the time spent waiting to be and being milked, time spent drinking water, and other non-productive related activity.

The social and grooming behavior of the focal cows was observed and recorded from the video recordings for a continuous 4-h period per day for 3 d (for 12 h total) (5, 8) after regrouping. Talebi et al. (8) observed focal cows from video recording for 3 h after feed delivery; however, after plotting preliminary data from the current study, a continued high occurrence of behavioral interactions was observed for both treatment groups continuing into the fourth hour after feed delivery; therefore, it was decided to observe these behavior for 4 h after feed delivery. Focal cows were observed immediately after joining the new group on d 1 and were observed immediately after feed delivery time on d 2 and 3 after regrouping. Cows on the control treatment were fed daily at 09:32 h ± 43:18 min, whereas cows on the CE treatment were fed daily at 09:57 h ± 27:56 min. Using the ethogram in Table 2, the following behaviors were recorded: displacement from the feed bunk, displacement from the stall, displacement in the alley, head butting, threatening, head-to-head contact, allogrooming, self-grooming, use of the brush, and scratching against the pen (against fixtures in the pen). Additionally, behavior events were distinguished as either actor (e.g., the focal cow was the one initiating the event) or reactor (e.g., the focal cow was the one receiving the event). Allogrooming (as actor and reactor) and self-grooming behavior were recorded as a single event if it was uninterrupted or interrupted by <20 s and then resumed; if the interruption lasted more than 20 s before being continued it was considered as two grooming events (31). All behavior events recorded were summarized as the frequency of events/d (31). Furthermore, to determine the overall effect of treatments on behavior, we summed some of the behavior variables together, including total displacements (displacements from the stall, feed bunk, and alley), aggression (head butting, threatening, head-to-head contact), and grooming (allogrooming, self-grooming, use of the brush, and scratching against the pen), both as actor and reactor. Further, the total displacements and aggression were summed together as the total competitive behavior (both as actor and reactor). Video analysis was performed by two observers (who were not able to be blinded to the treatments), after establishing an inter-observer reliability of κ ≥ 0.73 between the two observers.

**Table 2:** An ethogram of behaviors recorded from video observations.

**Behavior**	**Description**
**Event (actor**^**a**^ **and reactor**^**b**^**)**	
Displacement from the feed bunk^c^	A cow (actor) uses her head, shoulders, or flank to aggressively contact another cow (reactor), causing her to retreat (withdrawing her head) from the feed bunk
Displacement from the stall^d^	A cow (actor) uses her head, shoulders, or flank to aggressively contact with another cow (reactor), causing her to completely retreat from the stall
Displacement in the alley^e^	A cow (actor) uses her head, shoulders, or flank to aggressively contact with another cow (reactor), causing her to retreat to another place
Head butting^f^	When a cow (actor) uses her head to make contact with another cow (reactor)
Threatening^g^	When a cow (actor) chases or approaches another cow (reactor), causing this cow to withdraw from her current location
Head-to-head contact^h^	When a cow (actor) initiates contact using the front of her head in the direction of another cow and the other cow (reactor) retributes by also contacting the actor with the front of her head
Allogrooming^i^	When a cow (actor) initiates a grooming event on another cow (reactor)
**Event (actor** ^**a**^ **)**	
Self-grooming^j^	When a focal cow initiates a grooming event on herself
Use of the brush^k^	When a focal cow makes repetitive contact with their head or body against a brush
Scratching^l^	When a focal cow makes repetitive contact with their head or body against a fixture of the pen (e.g., a bar or a gate)
**Summed behaviors (actor** ^**a**^ **)**	
Displacement	Sum of displacements from the feed bunk, displacements from the stall, and displacements in the alley
Aggression	Sum of threatening, head butting and head-to-head events
Grooming	Sum of allogrooming, self-grooming, use of the brush, and scratching
**Summed behaviors (reactor** ^**b**^ **)**	
Displacement	Sum of times that the focal cows was displaced from the feed bunk, stalls, and in the alley
Aggression	Sum of times that the focal cows received an aggression (threatening, head butting and head-to-head) event
**Total summed behaviors**	
Total competitive behavior actor	Sum of all actor displacements and aggression
Total competitive behavior reactor	Sum of all reactor displacements and aggression

a *Actor, Individual focal cow initiates the behavior event*.

b *Reactor, Individual focal cow that receives the behavior event*.

c *Displacement from the feed bunk (26)*.

d *Displacement from the stall (27)*.

e *Displacement from the alley (28)*.

f *Head butting (29)*.

g *Threatening (29)*.

h *Head-to-head contact (29)*.

i *Allogrooming (5, 29)*.

j *Self-grooming (5, 29)*.

k *Use of the brush (27)*.

l *Scratching (30)*.

### Feed Sampling Analysis

Samples of each TMR (control and CE diets) were collected in duplicate 3x/ week. Orts (refusal) samples from each free-stall pen were taken 3x/week. In addition, orts from focal cows were collected 3x/week while the cows were in the tie-stall area. Focal cow orts in the tie-stall were weighed daily, and free-stall pen orts were weighed 3x/week across the study period. Upon feed sampling, all samples were immediately frozen at −20°C until further analysis. Samples were thawed for 1 d prior to being dried. All samples were oven-dried at 55°C for 48 h for DM analysis. Samples of TMR diets to be analyzed for nutrient content were ground by passing through a 1-mm screen (Model 4 Wiley Laboratory Mill, Thomas Scientific, Swedesboro, NJ). Ground samples were sent to A & L Laboratory Services Inc. (London, ON, Canada) for chemical analyses (Table 1) of ash [550°C; AOAC International, (32): method 942.05], ADF [AOAC International (32): method 973.18], NDF with heat-stable α-amylase and sodium sulfite [AOAC International, (32): method 2002.04], CP [N × 6.25; AOAC International, (32): method 990.03; Leco FP-628 Nitrogen Analyzer, Leco, St. Joseph, MI], starch [heat-stable amylase and amyloglucosidase; AOAC International, (32): method 996.11], fat [using pet ether, AOAC International, (32): method 920.39], lignin (using ADF residue and H_2_SO_4_), and minerals (using aquaregia digestion inductively coupled plasma atomic emission spectroscopy), and calculation of TDN and net energy [using NRC (18) equations].

### Milk Yield Data

Milk yield, at each milking, was measured and recorded for all focal cows throughout the study using the parlor milk weighing system and summarized on a daily basis (kg/d). Body weight (BW) and BCS were recorded for all cows in the free-stalls, at each milking, across the study period using automated scale and automated BCS camera (DeLaval BCS; Delaval, Tumba, Sweden), as validated by Mullins et al. (33), both placed on exit from the milking parlor. One value per cow per day was calculated by averaging across the afternoon and morning milkings.

### Statistical Analyses

All data were summarized on a daily basis with day beginning at 10:00 h and ending at 09:59 h the next day. All statistical analyses were conducted using SAS 9.4 software (34). For all analyses, outcomes were considered significant at *P* ≤ 0.05 and tendencies at 0.05 < *P* ≤ 0.10. Before analysis, all data were screened for normality and outliers using the UNIVARIATE procedure of SAS: milk yield, rumination time, lying time, idle standing time, and feeding time were normally distributed. Recorded behavior traits (Table 2) were right-skewed (due to a large number of zero values) and transformed, to achieve normality, by taking the natural logarithm +1. In preliminary analyses of the behavioral responses of cows after regrouping, we detected a treatment by parity interaction; therefore, to better address our hypotheses, we proceeded to analyze the treatment effect for each response variable within parity group (primiparous and multiparous).

Individual cow DMI and efficiency of milk production data were only collected in the baseline period. These data were analyzed in repeated measures mixed-effect linear regression models (using the MIXED procedure of SAS), treating day as a repeated measure. Fixed effects in the models included treatment (control or CE), day (d −7, −6, −5, −4, −3, −2, and −1 prior to regrouping), and the interaction between treatment and day. Cow was included as a random effect. The first-order autoregressive covariance structure was used for both models based on best fit, according to the lowest Bayesian information criterion values.

For milk yield, rumination time, and lying time, a preliminary analysis was conducted to verify there were no significant effects of day, or treatment by day interactions, within the baseline period. Given none, a baseline average of the 7 d before regrouping was generated, with d 1 representing the day that the focal cow was regrouped. These data were analyzed in repeated measures, mixed-effect linear regression models (using the MIXED procedure of SAS), treating day as a repeated measure. Models were specifically built to test whether the outcome variables after regrouping differed from their baseline average. Fixed effects in the models included treatment (control or CE), day (baseline average and d 1, 2, 3, 4, 5, 6, and 7 after regrouping), and the interaction between treatment and day. Cow was included as a random effect. The first-order autoregressive covariance structure was used for all models based on best fit, according to the lowest Bayesian information criterion values. Body weight, BCS, DIM, and 7-d average milk yield (of the focal cows) before entering the trial were all tested as covariates in the model. Primiparous CE cows produced 33.7 ± 0.62 kg/d of milk on average before being enrolled in the study (i.e., 7 d before being moved to the tie-stall), whereas primiparous control cows produced 30.2 ± 0.67 kg/d of milk before the study. Multiparous CE cows produced 36.4 ± 0.65 kg/d of milk, and multiparous control cows produced 41.8 ± 0.81 kg/d of milk before the study. No interactions between these covariates and treatment were detected. To test the hypothesis that cows changed their behavior (i.e., lying and rumination) and milk yield following regrouping, differences between days after regrouping and their baseline average, within treatment, were compared.

Feeding time and idle standing time data were analyzed in repeated measures mixed-effect linear regression models (using the MIXED procedure of SAS), treating day as a repeated measure. Fixed effects in the models included treatment (control or CE), day (d 1, 2, 3, 4, 5, 6, and 7 after regrouping), and the interaction between treatment and day. Cow was included as a random effect. The first-order autoregressive covariance structure was used for both models based on best fit, according to the lowest Bayesian information criterion values. Body weight, BCS, and DIM were tested as covariates in the model. No interactions between these covariates and treatment were detected. When treatment by day interactions were detected, differences between treatments were compared by day after regrouping using the PDIFF option in the LSMEANS statement.

Social behavior variables analyzed included displacement from the feed bunk, displacement from the stall, displacement in the alley, head butting, threatening, head-to-head contact, allogrooming, total displacements, total aggression, total grooming, and total competitive behavior, all of these variables were assessed for both actors and reactors, and self-grooming, use of the brush, and scratching against the pen were assessed only as a one-way event. These data were analyzed in repeated measures mixed-effect linear regression models (using the MIXED procedure of SAS), treating day as a repeated measure. Fixed effects in the models included treatment (control or CE), day (d 1, 2, and 3 after regrouping), and the interaction between treatment and day. Cow was included as a random effect. The first-order autoregressive covariance structure was used for all models based on best fit, according to the lowest Bayesian information criterion values. Body weight, BCS, and DIM were tested as covariates in the model. No interactions between these covariates and treatment were detected. When treatment by day interactions were detected, differences between treatments were compared by day after regrouping using the PDIFF option in the LSMEANS statement.

## Results

### Dry Matter Intake

In the baseline period (7 d in tie-stall area prior to regrouping) primiparous control cows consumed 24.5 ± 0.56 kg/d of DM, and primiparous CE cows consumed 24.8 ± 0.56 kg/d of DM. Multiparous cows that were fed the control diet consumed 30.2 ± 0.53 kg/d of DM and multiparous cows that were fed the CE diet consumed 29.5 ± 0.56 kg/d of DM in the baseline period. No differences were detected for DMI in the baseline period between the CE and control focal cows (for either primiparous or multiparous cows; *P* ≥ 0.37). Across the whole study period, the DMI of the free-stall (non-focal and focal) control cows averaged 27.1 ± 2.3 kg/d (mean ± SD), whereas, for the free-stall CE cows, it averaged 26.7 ± 2.7 kg/d.

### Rumination Behavior

Baseline rumination time of primiparous control and CE cows was 528.4 ± 21.8 and 485.9 ± 21.8 min/d, respectively (Table 3). Primiparous cows fed the control diet exhibited decreased rumination time on d 1 (*P* = 0.003) and d 2 (*P* = 0.02) after regrouping compared with their own baseline (Table 3), whereas, primiparous cows on the CE diet spent more time ruminating on d 2 (*P* = 0.03), d 3 (*P* = 0.002), d 4 (*P* = 0.02), and d 7 (*P* = 0.03), and tended to have greater rumination time on d 5 (*P* = 0.09) after regrouping compared with their baseline period average.

**Table 3:** Effect of treatment on the rumination time (min/d) of primiparous and multiparous Holstein dairy cows after regrouping.

	**Baseline^**a**^**	**Day after regrouping**							**SEM**	***P*-value^**b**^**
		**1**	**2**	**3**	**4**	**5**	**6**	**7**		
**Primiparous**										
Control^c^	528.4	459.1^*^	477.6^*^	524.3	498.9	500.6	511.3	530.1	20.9	0.40
Citrus extract^d^	485.9	484.3	533.3^*^	556.3^*^	536.3^*^	523.3^†^	521.6	535.3^*^	20.9	
**Multiparous**										
Control^c^	484.6	517.7	524.8	540.0^†^	541.8^†^	564.0^*^	526.4	525.1	24.0	0.60
Citrus extract^d^	490.7	499.7	549.1^†^	565.3^*^	571.1^*^	556.6^*^	536.9	554.0^†^	25.4	

a *Baseline, Average of 7-d prior to regrouping*.

b *P-value for overall effect of treatment*.

c *Control, control TMR (control diet; n = 16; primiparous = 7; multiparous = 9)*.

d *Citrus extract, control TMR with 4 g/d of citrus extract (CE diet; n = 16; primiparous = 7; multiparous = 9)*.

* *Indicates difference from Baseline value within treatment at P ≤ 0.05*.

† *Indicates tendency for difference from Baseline value within treatment at 0.05 < P ≤ 0.10*.

During the baseline period, multiparous control and CE cows spent 484.6 ± 25.0 and 490.7 ± 26.4 min/d ruminating, respectively (Table 3). Rumination time was increased from their baseline period average on d 5 (*P* = 0.009) and tended to increase on d 3 (*P* = 0.07) and 4 (*P* = 0.06) for multiparous control cows (Table 3); whereas multiparous cows fed the CE diet had greater rumination time on d 3, 4, and 5 (*P* < 0.05), and had a tendency for increased rumination time on d 2 (*P* = 0.07) and 7 (*P* = 0.06) when compared with their baseline average (Table 3).

### Lying Behavior

During the baseline period, primiparous control and CE cows spent an average of 807.6 ± 47.1 and 776.6 ± 50.1 min/d lying down, respectively (Table 4). Compared with their baseline period, primiparous cows fed the control diet exhibited a reduction in lying time on d 1 (*P* = 0.002) and tended to have reduced lying time on d 2 (*P* = 0.06) and 3 (*P* = 0.06) following regrouping (Table 4). However, for primiparous cows fed the CE diet, no change was detected in lying time from their baseline period average for the 7 d following regrouping (Table 4).

**Table 4:** Effect of treatment on the lying time (min/d) of primiparous and multiparous Holstein dairy cows after regrouping.

	**Baseline^**a**^**	**Day after regrouping**							**SEM**	***P*-value^**b**^**
		**1**	**2**	**3**	**4**	**5**	**6**	**7**		
**Primiparous**										
Control^c^	807.6	593.9^*^	672.1^†^	673.4^†^	783.1	719.2	698.6	693.0	62.4	0.22
Citrus extract^d^	776.6	780.0	728.4	746.7	805.7	786.3	784.7	711.3	50.5	
**Multiparous**										
Control^c^	757.9	720.6	751.1	706.0	759.8	698.54	676.1	805.5	47.8	0.18
Citrus extract^d^	774.2	660.4^*^	660.7^*^	683.1	656.1^*^	661.33^*^	677.4^†^	703.1	42.8	

a *Baseline, Average of 7-d prior to regrouping*.

b *P-value for overall effect of treatment*.

c *Control, control TMR (control diet; n = 16; primiparous = 7; multiparous = 9)*.

d *Citrus extract, control TMR with 4 g/d of citrus extract (CE diet; n = 16; primiparous = 7; multiparous = 9)*.

* *Indicates difference from Baseline value within treatment at P ≤ 0.05*.

† *Indicates tendency for difference from Baseline value within treatment at 0.05 < P ≤ 0.10*.

Multiparous control and CE cows spent 757.9 ± 50.3 and 774.2 ± 46.4 min/d, respectively, lying down during the baseline period (Table 4). For multiparous control cows there was no detected reduction in lying time on any of the 7 d after regrouping compared with their own baseline lying time (Table 4), whereas, multiparous CE cows exhibited a reduction in time spent lying on d 1, 2, 4, and 5 (*P* < 0.05), and tended to have reduced lying time on d 6 (*P* = 0.09).

### Feeding Time and Idle Standing Time

**Figure 1:**
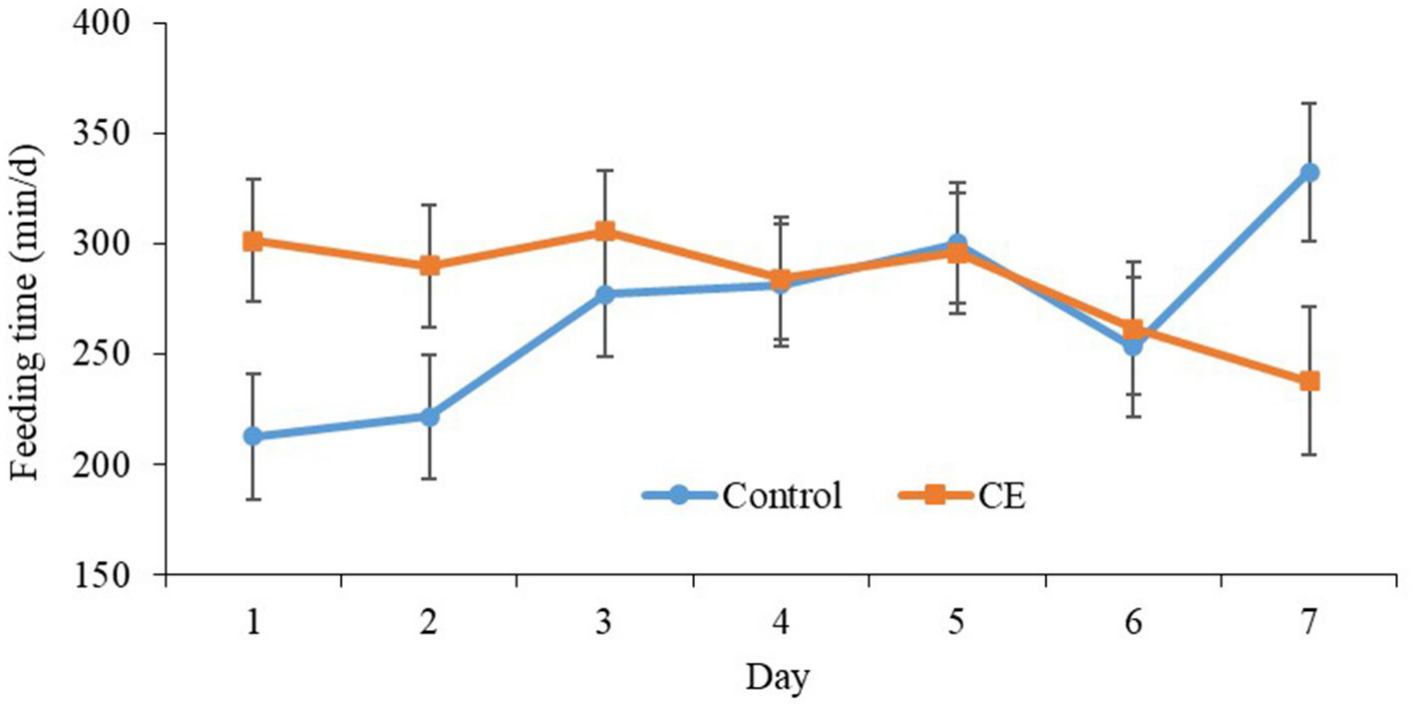
Effect of treatment on feeding time (min/d) (mean ± SE) of primiparous lactating cows: (1) control TMR (control diet; *n* = 7) or (2) control TMR with 4 g/d of citrus extract (CE diet; *n* = 7). Day, day following regrouping.

Primiparous control focal cows spent, on average, 268.5 ± 20.5 min/d at the feed bunk, whereas, primiparous CE cows spent 282.4 ± 20.4 min/d in the 7 d after regrouping (Table 5). Furthermore, multiparous focal cows that were fed CE spent, on average, 284.78 ± 14.7 min/d at the feed bunk, whereas, multiparous focal cows that were fed the control diet spent 309.4 ± 14.8 min/d at the feed bunk. A treatment × day interaction was detected for primiparous focal cow feeding time (*P* = 0.04, Figure 1). Primiparous focal cows that were fed CE had greater feeding time on d 1 (*P* = 0.03) and tended to have greater feeding time on d 2 (*P* = 0.09; Table 5) compared with primiparous control focal cows after regrouping. Furthermore, primiparous cows that were fed the control diet spent more time at the feed bunk on d 7 (*P* = 0.04; Table 5; Figure 1) than primiparous CE cows after regrouping. No difference was detected for time spent at the feed bunk between multiparous control and CE focal cows in the 7 d after regrouping (Table 5).

**Table 5:** Effect of treatment on the feeding time (min/d) of primiparous and multiparous Holstein dairy cows after regrouping.

	**Day after regrouping**							**SEM**	***P*-value^**a**^**
	**1**	**2**	**3**	**4**	**5**	**6**	**7**		
**Primiparous**									
Control^b^	212.7^d^	221.7*^†^*	277.3	281.4	300.3	253.4	332.4^d^	29.1	0.63
Citrus extract^c^	301.4^e^	290.0*^†^*	305.7	284.3	295.7	261.8	238.0^e^	28.8	
**Multiparous**									
Control^b^	280.3	311.7	302.1	321.5	339.2	287.4	323.8	20.4	0.24
Citrus extract^c^	263.0	275.4	291.4	288.6	285.9	299.4	289.7	20.0	

a *P-value for overall effect of treatment*.

b *Control, control TMR (control diet; n = 16; primiparous = 7; multiparous = 9)*.

c *Citrus extract, control TMR with 4 g/d of citrus extract (CE diet; n = 16; primiparous = 7; multiparous = 9)*.

d, e *Values within the same column (within parity group) with different superscripts indicates difference between treatments, within day, at P ≤ 0.05, given an overall treatment x day interaction (P = 0.04)*.

† *Indicates (within parity group) tendency for difference between treatments, within day, at 0.05 < P ≤ 0.10, given an overall treatment x day interaction (P = 0.04)*.

Mean idle standing time for primiparous control and CE focal cows was 504.1 ± 49.6 and 371.1 ± 38.8 min/d, respectively, whereas multiparous control focal cows spent 388.73 ± 43.4 min/d standing idle and multiparous CE focal cows spent 484.9 ± 38.0 min/d standing idle (Table 6). Primiparous CE cows spent less time standing idle (min/d) than primiparous control cows in the 7 d after regrouping (Table 6). Furthermore, no difference in idle standing time (min/d) was detected between multiparous CE and control cows in the 7 d after regrouping (Table 6).

**Table 6:** Effect of treatment on the idle standing time (min/d) of primiparous and multiparous Holstein dairy cows after regrouping.

	**Day after regrouping**							**SEM**	***P*-value^**a**^**
	**1**	**2**	**3**	**4**	**5**	**6**	**7**		
**Primiparous**									
Control^b^	685.25	549.00	534.58	386.75	447.69	488.84	436.70	81.10	0.04
Citrus extract^c^	352.83	415.17	383.67	329.67	334.00	367.38	414.76	60.61	
**Multiparous**									
Control^b^	470.66	362.82	367.48	358.08	418.48	411.92	331.68	56.04	0.11
Citrus extract^c^	537.24	528.14	457.46	477.52	484.98	462.75	446.07	47.79	

a *P-value for overall effect of treatment*.

b *Control, control TMR (control diet; n = 16; primiparous = 7; multiparous = 9)*.

c *Citrus extract, control TMR with 4 g/d of citrus extract (CE diet; n = 16; primiparous = 7; multiparous = 9)*.

### Social Behavior

The effect of treatment on the social behavior of primiparous focal cows is reported in Table 7. As compared with the primiparous control cows, the primiparous CE focal cows displaced other cows less often in the alley and had less total competitive behavior as actor (displacement and aggression variables summed) (Table 7). Those cows also tended to head butt other cows less often in the pen, to initiate less head-to-head contact, to displace fewer cows from all areas of the pen (feed bunk, free stalls, and alley), and engage in less aggression across all 3 d after regrouping compared to the primiparous control cows (Table 7).

**Table 7:** Effect of treatment on the behavior (#/d; natural log + 1)^a^ of primiparous Holstein dairy cows after regrouping (mean ± SE).

	**Treatment (T)**		***P*** **-value** ^**b**^		
**Variable**	**CON^**c**^**	**CE^**d**^**	**Treatment**	**Day**	**T × D**
**Actor**					
Displacement from the feed bunk	0.86 ± 0.24	0.64 ± 0.23	0.51	0.42	0.93
Displacement from the free stall	0.32 ± 0.14	0.28 ± 0.14	0.86	0.64	0.62
Displacement in the alley	1.12 ± 0.17	0.54 ± 0.16	0.02	0.13	0.43
Head butting	1.39 ± 0.23	0.75 ± 0.22	0.06	0.002	0.16
Threatening	0.65 ± 0.25	0.24 ± 0.25	0.27	0.006	0.39
Head-to-head contact	1.14 ± 0.22	0.51 ± 0.21	0.06	0.001	0.92
Allogrooming	0.33 ± 0.12	0.44 ± 0.11	0.47	0.64	0.68
Displacement^e^	1.62 ± 0.28	0.89 ± 0.27	0.08	0.13	0.94
Aggression^f^	1.92 ± 0.33	0.96 ± 0.32	0.06	<0.001	0.73
Total competitive behavior^g^	2.34 ± 0.35	1.32 ± 0.34	0.03	0.003	0.94
**Reactor**					
Displacement from the feed bunk	1.78 ± 0.26	2.42 ± 0.25	0.10	0.47	0.16
Displacement from the free stall	0.45 ± 0.17	0.51 ± 0.17	0.82	0.35	0.02
Displacement in the alley	1.58 ± 0.23	1.14 ± 0.22	0.19	0.01	0.66
Head butting	2.12 ± 0.34	1.87 ± 0.33	0.60	0.002	0.05
Threatening	2.06 ± 0.28	2.05 ± 0.28	0.97	<0.001	0.008
Head-to-head contact	1.09 ± 0.21	0.96 ± 0.20	0.68	0.01	0.62
Allogrooming	0.50 ± 0.15	0.54 ± 0.15	0.85	0.54	0.42
Displacement^h^	2.45 ± 0.23	2.73 ± 0.22	0.38	0.04	0.22
Aggression^i^	2.84 ± 0.29	2.80 ± 0.29	0.90	<0.001	0.002
Total competitive behavior^j^	3.30 ± 0.27	3.47 ± 0.27	0.67	0.002	0.007
Scratching against the pen	0.65 ± 0.23	0.70 ± 0.23	0.90	0.51	0.51
Self-grooming	1.74 ± 0.27	1.71 ± 0.26	0.93	0.22	0.59
Brush	0.67 ± 0.21	0.86 ± 0.20	0.52	0.007	0.51
Grooming^k^	2.28 ± 0.23	2.41 ± 0.23	0.70	0.92	0.10

a *All behavior variables (individual and summed) were log-transformed (natural log + 1), given that they did not meet the assumption of normality*.

b *P-values are provided for the effects of Treatment (T), Day (D), and T × D*.

c *CON, control TMR (control diet; n = 7)*.

d *CE, control TMR with 4 g/d of citrus extract (CE diet; n = 7)*.

e *Sum of displacement variables (feed bunk, free stalls, alley) as actor*.

f *Sum of threatening, head butting, and head-to-head as actor*.

g *Total competitive behavior variable is all displacements and aggression summed as actor*.

h *Sum of displacement variables (feed bunk, free stalls, alley) as reactor*.

i *Sum of threatening, head butting, and head-to-head as reactor*.

j *Total competitive behavior variable is all displacements and aggression summed as reactor*.

k *Sum of allogrooming (actor and reactor), self-grooming, scratching agains the pen, and brush*.

**Figure 2:**
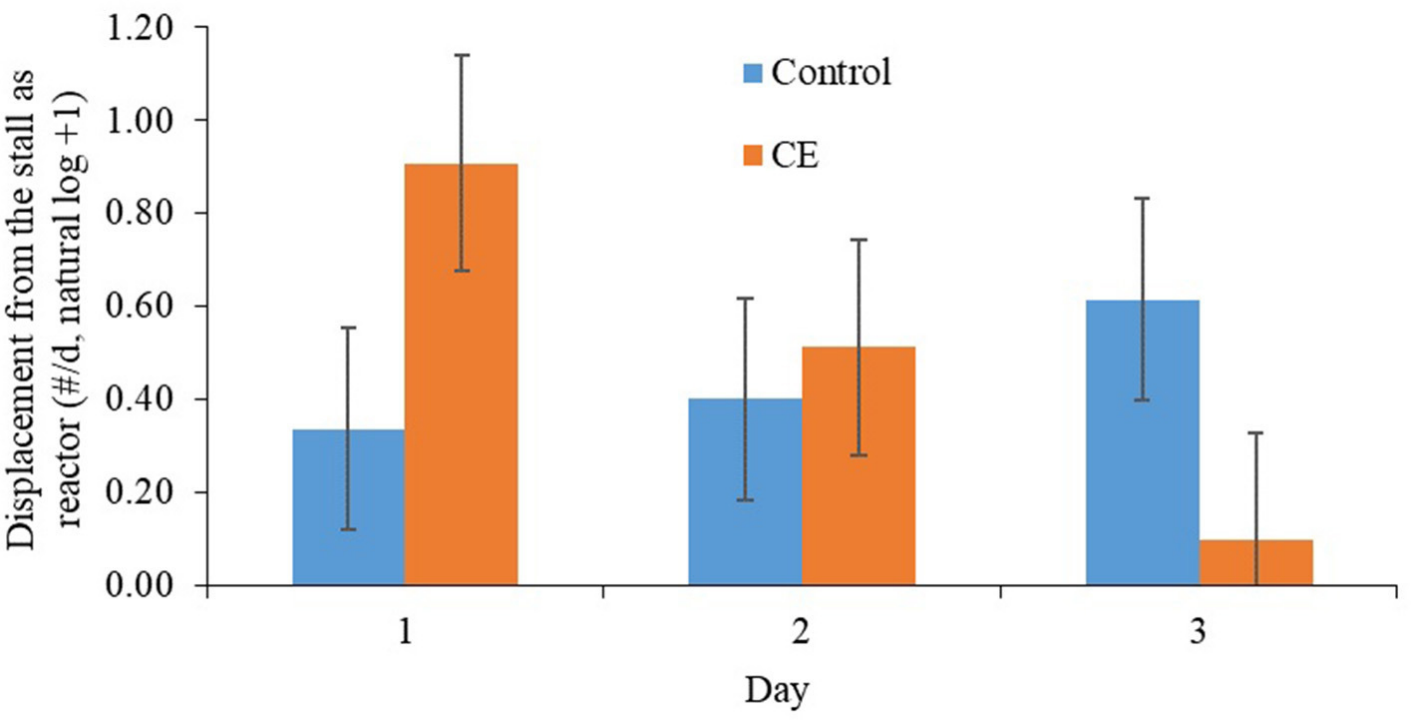
Effect of treatment on displacement (as reactor) from the stall (#/d, natural log +1; mean ± SE) of primiparous lactating cows: (1) control TMR (control diet; *n* = 7) or (2) control TMR with 4 g/d of citrus extract (CE diet; *n* = 7). Day, day following regrouping.

Primiparous focal cows that were fed the CE diet tended to be displaced more frequently from the feed bunk than primiparous focal cows that were fed the control diet after regrouping (Table 7). A treatment × day interaction was detected for displacements from the stall as reactor (Table 7), whereby primiparous CE focal cows tended be displaced from the stall more often on d 1 (*P* = 0.09) than primiparous control focal cows after regrouping (Figure 2). A treatment by day interaction was detected (*P* ≤ 0.1; Table 7) for threatening (reactor), head butting (reactor), aggression (reactor), grooming, and total competitive behavior (reactor) for primiparous focal cows; however, no differences between treatments within day were detected for any of these variables. We did not detect any differences between treatments for allogrooming, self-grooming, use of the brush, and scratching against the pen after regrouping.

The effect of treatment on social behavior of multiparous focal cows is reported in Table 8. Multiparous CE focal cows were head butted more frequently than multiparous control focal cows in the 3 d after regrouping (*P* = 0.03). A tendency for treatment by day was detected for displacements from the free stall (Table 8). Multiparous focal cows that were fed the CE diet displaced fewer cows from the free stalls on d 1 after regrouping (*P* = 0.001; data not shown) than multiparous focal cows that were fed the control diet.

**Table 8:** Effect of treatment on the behavior (#/d; natural log + 1)^a^ of multiparous Holstein dairy cows after regrouping (mean ± SE).

	**Treatment (T)**		***P*** **-Value** ^**b**^		
**Variable**	**CON^**c**^**	**CE^**d**^**	**Treatment**	**Day**	**T × D**
**Actor**					
Displacement from the feed bunk	1.21 ± 0.31	1.38 ± 0.31	0.70	0.60	0.97
Displacement from the free stall	0.33 ± 0.08	0.18 ± 0.08	0.23	0.02	0.08
Displacement in the alley	0.96 ± 0.16	1.05 ± 0.17	0.69	<0.001	0.36
Head butting	1.28 ± 0.23	1.66 ± 0.23	0.26	<0.001	0.23
Threatening	0.72 ± 0.18	0.92 ± 0.18	0.45	0.002	0.99
Head-to-head contact	0.90 ± 0.16	0.78 ± 0.16	0.62	<0.001	0.42
Allogrooming	0.42 ± 0.13	0.19 ± 0.13	0.23	0.42	0.91
Displacement^e^	1.78 ± 0.26	1.90 ± 0.27	0.74	0.01	0.85
Aggression^f^	1.84 ± 0.23	2.08 ± 0.24	0.48	<0.001	0.19
Total competitive behavior^g^	2.49 ± 0.25	2.65 ± 0.26	0.66	<0.001	0.49
**Reactor**					
Displacement from the feed bunk	1.63 ± 0.30	1.96 ± 0.30	0.44	0.36	0.18
Displacement from the free stall	0.30 ± 0.10	0.48 ± 0.11	0.23	0.01	0.28
Displacement in the alley	1.02 ± 0.22	1.28 ± 0.22	0.41	0.01	0.60
Head butting	1.48 ± 0.21	2.14 ± 0.21	0.03	<0.001	0.84
Threatening	1.25 ± 0.22	1.36 ± 0.23	0.71	<0.001	0.64
Head-to-head contact	0.84 ± 0.21	0.82 ± 0.21	0.94	<0.001	0.15
Allogrooming	0.42 ± 0.17	0.44 ± 0.17	0.94	0.18	0.54
Displacement^h^	1.98 ± 0.30	2.38 ± 0.30	0.35	0.002	0.27
Aggression^i^	2.19 ± 0.20	2.55 ± 0.21	0.22	<0.001	0.55
Total competitive behavior^j^	2.78 ± 0.23	3.16 ± 0.23	0.26	<0.001	0.52
Scratching against the pen	0.51 ± 0.16	0.59 ± 0.17	0.73	0.67	0.36
Self-grooming	1.30 ± 0.20	1.59 ± 0.20	0.30	0.74	0.75
Brush	0.52 ± 0.10	0.68 ± 0.10	0.27	0.02	0.88
Grooming^k^	1.93 ± 0.18	2.08 ± 0.19	0.58	0.50	0.71

a *All behavior variables (individual and summed) were log-transformed (natural log + 1), given that they did not meet the assumption of normality*.

b *P-values are provided for the effects of Treatment (T), Day (D), and T × D*.

c *CON, control TMR (control diet; n = 9)*.

d *CE, control TMR with 4 g/d of citrus extract (CE diet; n = 9)*.

e *Sum of displacement variables (feed bunk, free stalls, alley) as actor*.

f *Sum of threatening, head butting, and head-to-head as actor*.

g *Total competitive behavior variable is all displacements and aggression summed as actor*.

h *Sum of displacement variables (feed bunk, free stalls, alley) as reactor*.

i *Sum of threatening, head butting, and head-to-head summed as reactor*.

j *Total competitive behavior variable is all displacements and aggression summed as reactor*.

k *Sum of allogrooming (actor and reactor), self-grooming, scratching agains the pen, and brush*.

### Milk Yield

In the baseline period, primiparous control and CE cows produced 30.5 ± 1.42 and 35.0 ± 1.42 kg/d of milk, respectively (Table 9). Given similar DMI during that time period, primiparous cows fed the CE diet had greater efficiency of milk production across the baseline period (1.39 vs. 1.23 kg of milk/kg of DMI; *SE* = 0.049; *P* = 0.04). Even though we controlled for the milk yield of the cows prior to the study start, primiparous cows fed the CE diet had greater milk yield than primiparous control cows not only during the baseline period, but also across the 7 d after regrouping (Table 9). Primiparous control cows had decreased milk yield on d 1 after regrouping compared with their baseline average (*P* < 0.001; Table 9), whereas, primiparous cows fed the CE diet had decreased milk yield on d 1 (*P* = 0.01) and 7 (*P* = 0.04) after regrouping as compared with their baseline period average.

**Table 9:** Effect of treatment on milk yield (kg/d) of primiparous and multiparous Holstein dairy cows after regrouping.

	**Baseline^**a**^**	**Day after regrouping**							**SEM**	***P*-value^**b**^**
		**1**	**2**	**3**	**4**	**5**	**6**	**7**		
**Primiparous**										
Control^c^	30.5	28.1^*^	29.1	29.8	29.3	30.3	30.1	30.1	1.4	0.03
Citrus extract^d^	35.0	32.8^*^	33.8	34.6	33.5	33.5	34.0	32.9^*^	1.4	
**Multiparous**										
Control^c^	39.4	38.2	38.8	39.4	40.5	40.5	41.0	40.3	1.9	0.94
Citrus extract^d^	40.8	38.2^*^	38.0^*^	40.0	39.3	40.0	39.2	41.1	1.9	

a *Baseline, Average of 7-d prior to regrouping*.

b *P-value for overall effect of treatment*.

c *Control, control TMR (control diet; n = 16; primiparous = 7; multiparous = 9)*.

d *Citrus extract, control TMR with 4 g/d of citrus extract (CE diet; n = 16; primiparous = 7; multiparous = 9)*.

* *Indicates difference from Baseline value within treatment at P ≤ 0.05*.

During the baseline period, multiparous control and CE cows produced an average of 39.4 ± 1.87 and 40.8 ± 1.85 kg/d of milk, respectively (Table 9). No difference (*P* = 0.22) between multiparous control (1.32 ± 0.047 kg of milk/kg of DMI) and CE (1.41 ± 0.049 kg of milk/kg of DMI) cows was detected for efficiency of milk production during the baseline period. For multiparous cows fed the control diet, no difference from the baseline period average in milk yield was detected in the 7 d after regrouping (Table 9), whereas multiparous cows fed the CE diet had decreased milk yield on d 1 (*P* = 0.005) and 2 (*P* = 0.03) compared with their baseline.

## Discussion

Our objective was to determine if feeding a CE (derived from *C. sinensis*) would reduce the negative impact of regrouping of lactating dairy cows on behavior and milk production, particularly for those primiparous cows who would have been more naïve to such a social stressor. In previous research, supplementation of CE has been demonstrated to have anxiolytic effects in rats (12) and humans (35, 36). To address our objective, an experimental model was designed in which (focal) cows were moved from group (free-stall) housing to a tie-stall area to individually acclimate cows (for 7 d) to their respective treatment diets. Following this potentially stressful event, cows were then subjected to another stressful move by placing them into a new social group. Cows were regrouped with conspecifics that were on the same treatment diet, in order to maintain the focal cows on the same treatment and mimic what would happen in commercial settings. As hypothesized, supplementation of CE to primiparous cows led to a lower change in behavior (lying time, rumination time) and lesser competitive behavior in the 7 d after introduction into a new social group. For multiparous cows, however, CE supplementation was not consistently associated with any benefits to behavior or production after regrouping.

### Primiparous Cows

#### Feeding Time

Primiparous cows that were fed CE had greater feeding time on d 1 and tended to have greater feeding time on d 2 after regrouping as compared with the primiparous cows that were fed the control diet. Despite not being able to assess the change in feeding time upon regrouping of primiparous focal cows, the difference between the CE and control primiparous cows suggests that regrouping had a greater negative effect on the feeding time of primiparous control cows compared with the CE cows.

#### Lying Time

In our study, time spent lying down also decreased drastically on the first day after regrouping and returned to baseline after 3 d for the primiparous control focal cows. This is consistent with previous research that also detected a decrease in lying time on d 1 after regrouping (5). Moreover, von Keyserlingk et al. (5) suggested that focal cows were more unwilling to displace their pen mates to gain access to a preferred stall, thus, spending less time lying down following regrouping. Alternatively, in this current study, lying time was sustained in the 7 d after regrouping for the primiparous CE cows, again indicating that CE reduced the negative effect of regrouping. The sustained lying and greater feeding time immediately after grouping of the primiparous CE focal cows in our study may have contributed to their lesser idle standing time. Overall, this indicates that primiparous CE cows may have used their time more efficiently after regrouping as compared with the control primiparous cows.

#### Rumination Time

Inconsistent results have previously been reported for the effect of regrouping cows on rumination time. Hasegawa et al. (37) reported no change in rumination time after cows were regrouped. However, in that study the investigations of rumination time of the cows started 2 d after regrouping and, therefore, a drop in rumination may have been missed. Alternatively, in a study with dry cows fed with automated feed bins, rumination time was decreased on d 1 after regrouping (6). Those researchers suggested that the decrease in rumination time after regrouping was associated with decreased DMI following regrouping. We can theorize that in our study the sustained rumination time of the primiparous CE cows following regrouping was associated with greater feeding activity on d 1 and 2, and the sustained lying time (38), and potentially a change in DMI given its association with both greater feeding and rumination time (39). However, we were not able to measure individual DMI of the socially grouped focal cows, thus, challenging the ability to predict if the rumination time in our study was driven primarily by DMI (38).

#### Social Behavior

von Keyserlingk et al. (5) reported that focal cows were displaced more regularly at the feed alley by their pen mates in the 3 d following regrouping as compared with their baseline value. Schirmann et al. (6) also reported that focal cows displaced more cows at the feed bin on d 1 after regrouping, likely due to greater competition there. Although our study was not designed to assess any change in aggressive behavior from the baseline, we did detect differences in aggressive behaviors between the CE and control primiparous cows. Researchers have demonstrated that rats and pigs may react to social stressors by engaging in more aggressive behavior (40, 41). Alternatively, Nogues et al. (42) reported that some heifers avoided engaging in aggressive interactions, whereas, other heifers engaged in more aggressive interactions after regrouping. Interestingly, in that study, those heifers less willing to engage spent less time feeding and resting than the heifers that were more willing to engage in aggressive interactions to access important resources (i.e., feed bunk, lying stalls, waterers). These findings suggest that heifers undergoing social stress may vary in their engagement in aggressive behavior after regrouping, due to their individual characteristics. In the current study, primiparous cows that were fed CE tended to be displaced more frequently from the feed bunk and from the free stalls on d 1 after regrouping as compared with the primiparous control cows, potentially reflecting the fact that the primiparous CE cows spent more time eating at the feed bunk and lying in the free stalls. Thus, those cows would have been more susceptible to be displaced at those places than the primiparous control cows, who had greater idle standing time (away from the stalls and bunk) after regrouping. Potentially related to that greater idle standing time, those primiparous control cows displaced other cows more often in the alley, had more total social behaviors observed as actor (displacements and aggression variables summed), and tended to head butt other cows more often in the pen, to initiate more head-to-head contact, to displace more cows in the pen, and engage in more aggression than primiparous CE focal cows across all 3 d after regrouping. Greater social stress following regrouping may be a potential explanation for the greater frequency of these aggressive behaviors initiated by the primiparous control focal cows, as compared with the primiparous CE cows. Social stress can be defined as social events (regrouping, weaning, restraint) that trigger a psychological stress response, disturbing the homeostatic state of the animal (43). The present results support our hypothesis that feeding CE to cows would mitigate the social stress effects of moving cows into a new social group. Similarly, in previous research it has been demonstrated that CE will mitigate responses to social stress related to dietary transitions and situations causing anxiety (12, 17, 44).

Increased aggressive behavior of cows after regrouping may lead to more social stress, which can decrease milk yield (43). Similar to other studies (5, 37, 45), both primiparous CE and control focal cows experienced a reduction in milk yield on the first day following regrouping. However, despite controlling for pre-trial milk yield, the primiparous CE cows had greater milk yield across both the baseline period and the 7 d after regrouping as compared with the primiparous cows fed the control diet. The greater milk yield in the primiparous CE cows as compared with primiparous control cows may have been due to differences in behavior elicited by the treatment. For example, the CE cows had overall greater feeding time immediately after grouping, less idle standing time, and initiating lesser aggressive interaction than primiparous control cows after regrouping. Further, those primiparous CE cows also had sustained rumination and lying time compared with their baseline as compared to the primiparous control cows after regrouping. Ensuring cows have sufficient time to devote to these behaviors is important for the maintenance of milk production (39, 46). We theorize that primiparous cows supplemented with CE had reduced anxiety, therefore reducing aggressive behavior and allowing those cows to spend more time at productive behaviors (i.e., feeding, lying, ruminating, etc.) following regrouping. It is also possible that the CE supplementation helped those primiparous cows cope with stress during the baseline period (and thus produce more milk), as movement to the tie-stall area would have already been stress inducing in all the cows.

### Multiparous Cows

No difference in feeding time was detected between multiparous CE and control focal cows after regrouping. Further, rumination time was sustained on d 1 and increased on some days for both multiparous CE and control focal cows after regrouping. The multiparous CE focal cows had more days with increased rumination time after regrouping as compared with their baseline than multiparous control cows, suggesting that CE may have also had some positive effect on the multiparous cows. However, the multiparous CE cows also had decreased time spent lying on most days after regrouping, whereas, the multiparous control cows sustained their lying time across the 7 d of regrouping. These results are difficult to interpret given that rumination and lying time are often positively correlated (38). Citrus extract supplementation had little effect on the social behavior of the multiparous cows, with the multiparous CE focal cows being head butted more often than multiparous control cows across the 3 d following regrouping and the multiparous control cows displacing more cows from the stall on d 1. Furthermore, the multiparous CE cows had reduced milk yield on d 1 and 2 after regrouping compared with their baseline. However, this result does not correspond with other measures of social stress at that time (e.g., changes in rumination, lying time, idle standing, feeding behavior, and social behavior). Therefore, the cause of this reduction milk yield after regrouping detected in the multiparous CE cows in unknown and, thus, needs to be evaluated in further research.

### Response of Primiparous vs. Multiparous Cows

We hypothesize that the lesser CE effect observed for the multiparous focal cows may be due to the lesser challenging effect that regrouping has on multiparous cows. Overall, the primiparous control cows experienced a reduction in rumination time, lying time, and milk yield compared with their baseline after regrouping. In contrast, the multiparous control cows did not experience as substantial a decrease in rumination time, lying time, and milk yield after regrouping as compared with their baseline. This is not entirely surprising, given that primiparous cows have been reported to have less competitive success at the feed bunk than multiparous cows (47), to have greater fecal cortisol when regrouped individually than multiparous cows regrouped individually (10), and cope less well with postpartum regrouping than multiparous cows (11). Thus, these studies indicate that primiparous cows are more susceptible to social stress than multiparous cows. Further, this would be enhanced in our study by the fact that the multiparous focal cows would have previously been exposed to more social stressors, and other cows, than the primiparous focal cows prior the start of the trial. This is due to the cows in our research station being handled, moved, and regrouped more often (due to involvement in research projects) than they would typically be under commercial management. As result, the focal multiparous cows would have also been previously exposed to more of their conspecifics, and thus may have had more pre-existing, established social relationships with the other cows in the groups they were introduced within. Thus, those multiparous cows may have been more acclimated to the stress associated with regrouping as well as potentially knew more of their new pen mates. Alternatively, the primiparous focal cows were more naïve to that type of stressor as well as more likely unknown to their new pen mates. This may also explain the lack of consistent response across behaviors, between treatments, within the multiparous cows. Previous exposure to social stress may have helped some of those multiparous cows cope with regrouping, while for other cows it is possible that previous social stress actually reinforced a greater negative response.

### Limitations and Future Studies

To our knowledge, this work is the first to study the effects of a CE on the behavior and production of lactating cows after regrouping. While effects were demonstrated, these need to be interpreted in light of limitations associated with the experimental model used, and corroborated in further studies.

Primiparous CE cows were subjected to more aggression within their host pen (displacements at the feed bunk and free stalls) from their pen mates than primiparous control cows. While, as discussed, this could be related to them spending more time engaged in eating and lying behavior, this could also have been due to an overall difference in social structure between the host pens, as result of experimental design. It is difficult to assess the extent of the effect that social structure had on the results of this study. Initially, the cow composition of both host pens were similar (i.e., in relation to days in milk, parity, body size, milk production, etc.). Beyond that, no accounting of social structure was made; thus, a random social structure effect was applied for both host pens. Over time, as cows were introduced and removed from each pen (on a weekly basis) the social dynamic of those pens would have varied. While that variation over time may have minimized the potential confound of host pen social dynamic and treatment, it is however unknown if there were major social differences between those host pens, and if that had a major effect on the outcomes of this study. Further, as the dietary treatments were applied within each respective host pen (to all animals), it is possible that may have further influenced the behavior of the host pen cows. For example, the greater aggression experienced by the primiparous CE cows may have been a reflection of a change in behavior of their pen mates in response to that treatment. Another related limitation to this experimental design was the use of two pens (one for control cows and one for CE cows). While this was done to allow cows to remain exposed to the same treatment upon regrouping, as discussed earlier, there could have been unmeasured pen-related (location) effects that may have influenced the behavior of the cows. Thus, with these limitations in mind, future research is need to replicate these results, specifically through movement of focal cows (within treatment) into replicated hosts pens (that vary in social structure and location) to minimize these potentially confounding effects.

Related, another limitation of this study was that it was not possible for each focal cow to be introduced to the exact same social structure within treatment, due to one of the non-focal cows needing to leave its treatment pen each week upon entry of the new focal cow to be dried off. As such, each focal cow may have experienced a slightly different social structure after regrouping, increasing variability in the dynamic situation between focal cows within each treatment. An alternative would be to have each focal cow added to her host treatment pen, followed for 7 d, and then removed. In this way, the social dynamic of the 29 non-focal cows may have remained the same (more or less) for each host pen over the course of the study (although that may change with advancement into lactation), with the only change being the new focal cow introduced each week. In that case, and similar to that suggest above, replication of those host pens would be needed to minimize the confounding effect of pen and treatment.

As described earlier, to ensure that cows were acclimated to their respective treatment diets, focal cows were moved into a tie-stall area for 7 d before moving to the free-stall treatment pens. This experimental design may have accentuated the level of stress in all cows, as moving them from a free-stall area to tie-stall would have been stressful in itself. It is unknown if this stress influenced their behavior upon exposure to the subsequent stress of being moved into a new social group. It is possible that without that added stressor, a different treatment response would have been observed. Thus, future studies in this area should aim to study the effect of the CE on cows only exposed to a single relocation related stressor. In addition, further research is warranted to investigate the effects of CE supplementation on responses of dairy cows to other types of stressful situations, caused by either acute or chronic stressors.

## Conclusion

Results of this initial, preliminary study indicated that feeding CE to primiparous cows may have mitigated the negative effect of regrouping on their behavior. Specifically, primiparous CE cows had a lesser change in time spent lying and ruminating compared with their baseline after regrouping. Primiparous cows fed CE had greater feeding time immediately after regrouping as compared to primiparous control cows, and as a result of sustained lying time, the primiparous CE cows displayed less idle standing time. Overall, the primiparous control cows initiated a greater number of competitive interactions, whereas the primiparous CE cows received more aggressive interactions after regrouping. Although feeding CE reduced the negative effects of regrouping on primiparous cows, CE supplementation had little effect on the response of multiparous cows to regrouping. This suggests that the effect of regrouping tested herein may have been greater for more naïve primiparous cows compared to multiparous cows who would have been more experienced with social stressors. As this work is the first of its kind, these results need to be verified in further studies where potential confounding effects (e.g., pen social dynamics, pen location) are minimized. It is anticipated that this study will direct future studies investigating the use of olfaction sensory-based feed additives to mitigate social stress in dairy herds.

## Data Availability Statement

The raw data supporting the conclusions of this article may be made available, upon request, by the authors. Requests to access the datasets should be directed to the corresponding author.

## Ethics Statement

This animal study was reviewed and approved by University of Guelph Animal Care Committee (AUP#4131).

## Author Contributions

FP: conducted the study, collected data, analyzed data, and wrote this manuscript for submission. KD: collected data and analyzed data. RB: reviewed this manuscript for submission. TD: oversaw and designed the study, analyzed the data, and reviewed this manuscript for submission. All authors contributed to the article and approved the submitted version.

## Funding

This project was financially supported by Phodé (Terssac, France) and through a Natural Sciences and Engineering Research Council of Canada (NSERC; Ottawa, ON, Canada) Discovery Grant, as well as received support from the Ontario Agri-Food Innovation Alliance Research Program of the University of Guelph and the Ontario Ministry of Agriculture, Food, and Rural Affairs (Guelph, ON, Canada). Further, project equipment was supported by contributions from the Canadian Foundation for Innovation (CFI; Ottawa, Ontario, Canada) and the Ontario Research Fund (Toronto, ON, Canada).

## Conflict of Interest

The authors declare that the research was conducted in the absence of any commercial or financial relationships that could be construed as a potential conflict of interest.

## Publisher's Note

All claims expressed in this article are solely those of the authors and do not necessarily represent those of their affiliated organizations, or those of the publisher, the editors and the reviewers. Any product that may be evaluated in this article, or claim that may be made by its manufacturer, is not guaranteed or endorsed by the publisher.
